# Fishers' perception of the interaction between the South American sea lions and the Chinook salmon fishery in southern Chile

**DOI:** 10.1038/s41598-021-93675-x

**Published:** 2021-07-14

**Authors:** M. Sanguinetti, B. Cid-Aguayo, A. Guerrero, M. Durán, D. Gomez-Uchida, M. Sepúlveda

**Affiliations:** 1 Centro de Investigación y Gestión de Recursos Naturales (CIGREN), Universidad de Valparaíso, Gran Bretaña 1111, Playa Ancha, Valparaíso, Chile; 2 Facultad de Ciencias del Mar, Universidad de Valparaíso, Valparaíso, Chile; 3 Facultad de Ciencias Sociales, Universidad de Concepción, Concepción, Chile; 4 Núcleo Milenio INVASAL, Concepción, Chile; 5 Departamento de Sociología, Magister en Investigación Social y Desarrollo, Facultad de Ciencias Sociales, Universidad de Concepción , Concepción, Chile; 6 Departamento de Zoología, Facultad de Ciencias Naturales y Oceanográficas, Universidad de Concepción, Concepción, Chile

**Keywords:** Conservation biology, Environmental sciences

## Abstract

We studied how the South American sea lion (SASL, *Otaria flavescens*) interacts with the operation of an artisanal fishery of Chinook salmon, a non-native species in Chile, using a combination of biological and social approaches, including a valuation by fishers about this interaction. During austral summer of 2019, an observer onboard artisanal fishing boats characterized the attack behavior of SASLs to gillnet-captured Chinook salmon during 33 hauls and analyzed which factors may affect the intensity of attacks. To analyze the relationship between fishers and SASLs, a Likert scale about the perception and views about nature was applied. A total of 23 interviews—including 35 open and 16 closed questions—with fishers were conducted to describe how they perceived the interactions with SASLs. Interactions with SASLs were recorded in 35% of the fishing events and varied depending on both operational factors, such as the number of boats, as well as environmental factors, such as moon’s luminosity. Even though SASL interactions resulted in seven fish (~ 70 kg) damaged of a total catch of 2815 kg (2.5%) during the survey, boats with a damaged catch by SASL lost up to 11% of their revenue. This is consistent with 87% of the interviewed fishers who considered that the conflict with the SASL negatively impacts their activity and results in economic losses. A negative perception towards SASLs likely results from personal experience and revenue loss, even though impacts of SASL interactions at the scale of the entire fishery may be less important. While older fishers with less formal education have a productivist and instrumental focus, younger fishers with a more sustainable and conservationist view of fishing offer an opportunity to lead an improved local understanding of the relationship between salmon, SASLs, and humans.

## Introduction

Different species of salmonids were introduced in southern Chile, first to support recreational fisheries in the early 1900s and then for ranching and aquaculture-fishery purposes during the 1970s^1,2^. Chinook salmon (*Oncorhynchus tshawytscha*) is possibly one of the most successful salmonids after colonizing lakes and rivers in the northern and southern Patagonia during the 1980s^3,4^, establishing naturalized, migratory populations in multiple watersheds^1,5,6^. Several environmental impacts of this invasive species have been reported on native fishes as a result of direct, indirect and cascade effects, both in the ocean and in freshwater habitats^1^. In spite of the environmental impacts, the socioeconomic appreciation of Chinook salmon is high due to its economic, recreational, and culinary value, culminating in a difficult dilemma between the conservation of native biodiversity and the economic value of an invasive species^1,7^.

 La Barra is a small fishing village with a population of approximately 102 people located at the mouth of the Toltén River in southern Chile. Traditionally, the main economic activity in La Barra was the artisanal fishery of native marine species, mostly corvinilla (*Sciaena gilberti*) and sea bass (*Dicentrarchus labrax*), and to a lesser extent, catadromous species that inhabit the estuary; such as grey mullet (*Mugil cephalus*) and silverside (*Odontesthes bonariensis*). The artisanal fishery in this village is considered a family activity, since whole family groups are often directly or indirectly involved in this activity. Since 2010, an important change in the species community has affected the Toltén River; abundance of traditional native species diminished and large numbers of Chinook salmon were seen migrating upstream in route to spawning grounds^8^. This change in species community has transformed local fishing practices. Fishers now extract this abundant resource in the mouth of the river during the months of January and February, becoming the main economic activity for La Barra. In fact, for several families this invasive species constitutes its only annual income^8^. From 2010 until 2017 this was an illegal fishery. However, from 2017 to date La Barra fishers found a legal path to regulate and report Chinook salmon catches. With this new opportunity for economic development, La Barra fishers are now facing a new challenge: predation of gillnet-captured Chinook salmon by the South American sea lion (SASL, *Otaria flavescens*).

The SASL is a marine mammal (family Otariidae) widely distributed off South America in both the Atlantic and the Pacific Ocean basins^9,10^. In Chile, this species can be found in several coastal rookeries with an estimated abundance of nearly 130,000 individuals in the entire country^11,12^. It is classified as a ‘Least Concern’ by the Chilean regulation of wild species classification according to their Conservation categories, and protected by a 10-year ban from 2021 that prohibits harassment, capture, and harvest of SASLs. The SASL displays generalist and opportunist feeding habits, with a highly variable diet and easy adaptation to exploit the resources that are most widely available in their foraging environment^9,13^. Most of the species composing the diet of SASLs are of commercial importance^14^. This in turn has led to high levels of interaction with fishing activities throughout its distribution range^15–20^, because SASLs have learned to take advantage of prey concentrated and vulnerable inside fishing gear^18,21^. Interestingly, all aforementioned studies on SASL-fisheries interaction have focused on the predation of native species, but never on a non-native prey. To understand how a native predator may take advantage of this new prey resource and modify its trophic ecology and behavior is an interesting avenue for increasing our knowledge about the opportunistic behavior of this top marine predator.

Fishers and sea lions have a strained relationship. Several studies show that the negative interaction between both actors is due to fishers’ working areas largely overlap with SASL foraging areas^18,22^. Interaction includes: (1) sea lions taking fish from lines or nets with consequent gear damage or loss of the catch, (2) disturbance of fishing operations, and (3) sea lions mortality due to entanglement in fishing gear, or being intentionally killed by fishers^23^. Potential solutions to this conflict are more likely to succeed if they are pluralistic and include both a quantification of the problem and the human dimension associated with fishers’ perception of damage, defined as “a belief, whether rational or irrational, held by an individual, group, or society about the chance of occurrence of a risk (or any impact) or about the extent, magnitude, and timing of its effect(s)”^24–26^, on the interference by SASLs. According to Pont et al.^24^ and Oliveira et al.^26^, the relationship between fishers and sea lions not only relate to economic loss but also to an occasionally distorted perception of sea lions' damage. This is especially important considering that fishing involves an inherent interaction with the natural elements, and its success relies on natural processes and the wellbeing of the environment, as well as relying on the knowledge that fishers have of those cycles.

The classical concept of “valuation language”^27^ refers to the opinions, views and actions of people whose livelihood is directly linked with nature (e.g. farmers, gatherers and fishers). These people display an “environmentalism of the poor"- that is a deep understanding and appreciation of the environment. The environmentalism of the poor could be either a productivist-instrumental view of nature (i.e. nature is seen as a resource to be managed in order to obtain some benefit sustained over time), or a conservationist view of nature (i.e. nature as having a value by itself, regardless of its use by human beings). This “valuation language” perspective has been mostly used to understand the role of grass root movements in socio-environmental conflicts^28,29^, but has been rarely used to understand productive practices. Marten^30^ observed that fishers appreciate nature and reinforce conservation measures when they see them as being linked to a better fishing yield. Because environmental regulations (such as those protecting sea lions) that threaten their fishing activities and then their livelihood may encounter important resistance^24^, it is relevant to explore how local fishers perceive sea lions and more generally how they perceive nature, to understand and promote a more harmonious relationship among fish, sea lions and fishers. The inclusion of human dimensions into managing this conflict may help managers to guide actions to mitigate the problem^24^.

This study intends to characterize the interaction between the SASL and the Chinook salmon fishery at La Barra, including biological aspects and social implications, during Chinook salmon’s peak return to spawn at Toltén River. Specifically, we: (1) characterized the attack behavior of SASLs to gillnet-captured Chinook salmon; (2) evaluated the factors that affect the intensity of the attacks; (3) described how this interaction is perceived by fishers; and (4) documented the fishers’ opinion of the conflict by taking into account fishers’ relationship with nature. We hypothesized that socio-demographic variables, together with a broad view of nature affect the fishers’ perception about the relationship with SASLs. We adopted a symmetrical approach to the conflict that provides an understanding of the relationship between nature and culture as a process of coproduction, defined as the interactive change between social and natural dynamics, mediated by processes of labor and language^31–33^.

## Materials and methods

### Area and study period

**Figure 1:**
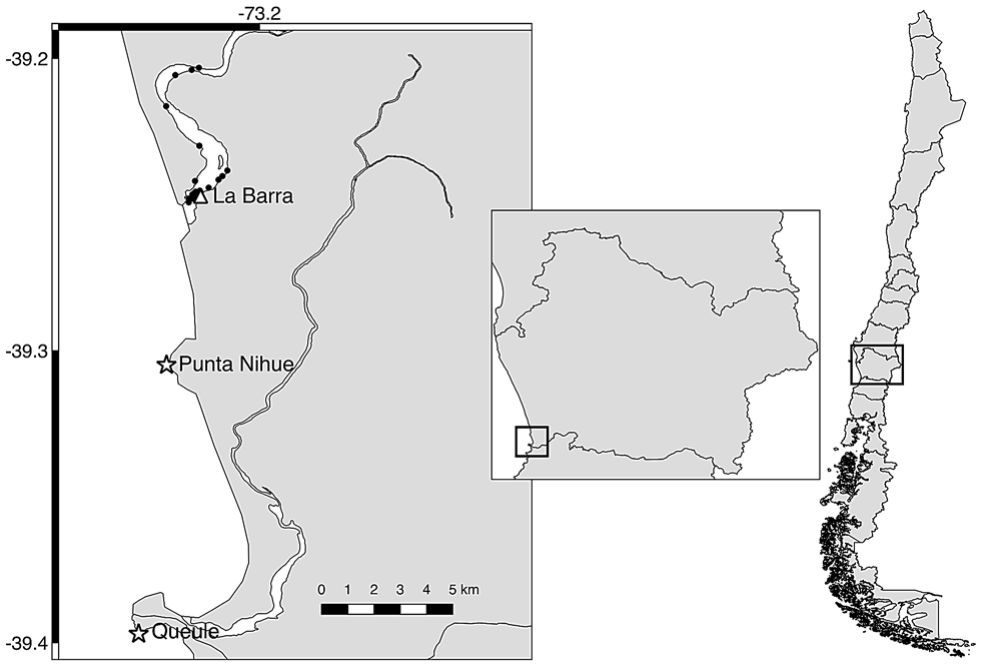
Map of the study area showing the location of the 33 hauls onboard artisanal fishing boats and the location of the two South American sea lion colonies close the area: Punta Nihue and Queule. Figures were done by QGIS software version 2.18 (http://www.qgis.org/).

This study was carried out in the artisanal fishing village of La Barra (39°14′ S, 73°13′ W), in southern Chile (Fig. 1). Only La Barra fishers  are authorized to capture Chinook salmon in the area located between the mouth of the Toltén River (from 39°15′ S) and the town of Toltén viejo (39°12′ S), a total distance of 6.8 km (Fig. 1).

Two colonies of SASL are close to the study area: Punta Nihue (39°18′ S; 73°14′ W) and Queule (39°23′ S; 73°14′ W), with an estimated abundance of 101 and 88 individuals, respectively^12^ (Fig. 1). None of these places are breeding colonies, although sea lions can be found in the rookeries throughout the year (M. Sepúlveda, pers. obs.).

A total of 22 fishing trips with 33 hauls onboard artisanal fishing boats were surveyed between January 25th and February 14th, 2019. This period was selected because the peak return of Chinook salmon to the Toltén River to spawn occurs during the austral summer months (January and February) and thus it is only fished during this period^8^. Fishing trips typically started during the evening at 20:00 and finished during the morning at 07:00. Observations were made by the same observer using 10 × 15 binoculars and the naked eye.

### Interaction with sea lions

During each haul, the following information was recorded: (1) geographic position using a GPS, (2) number and surface area of nets, (3) duration of the haul, (4) abundance and biomass of Chinook salmon caught, (5) immersion duration of the fishing net, (6) depth of point where fishing took place (information provided by the fishers), (7) number of SASLs observed within 20 m of the fishing vessel, a distance which provides a clear view of animals around the vessel^18^, (8) presence or absence of an interaction event, (9) number of sea lions interacting with the fishing vessel, (10) the moment of interaction (at the beginning, during or at the end of the fishing operation), (11) number of fish damaged by SASL attacks, (12) damage to the gillnets and (13) number of dead SASLs, differentiating intentional (i.e. by direct action by fishers) from accidental causes (due to entanglement in the net or during fishing operations). An interaction event was defined as when fish were damaged or taken from the gillnet, and/or the gillnet was damaged, as well as when the presence of SASLs caused a perturbation in the fishing operation^34^. We considered that a fish had been damaged by a sea lion when the scar had a characteristic semicircular shape and one or more SASLs were observed near the vessel^15^. Damage to gillnets was measured as the number of holes made by SASLs during the haul. All methods were carried out in accordance to national guidelines and regulations. As the study was non-invasive in nature, there was no need for the approval of the Ethics Committee of the University of Valparaíso.

Catch from all trips was standardized as Catch per Unit Effort (CPUE). CPUE was calculated as: *CPUE* = *C t*^−1^* s*^−1^, where *C* is the biomass of Chinook salmon (kg) captured per haul, *t* is the immersion duration of the gillnet (h), and *s* is the gillnet total surface (m^2^)^15^.

We applied each model as a generalized linear model (GLM) using Poisson distribution for counts variables (response 1: number of SASLs observed in the fishing area) and binomial distribution for presence/absence response variables (response 2: presence/absence of an interaction between SASL and the fishing operation). We used a model selection approach to determine which factors best explained the mentioned response variables. Due to the small sample size and large number of predictors, we run separate models combining 1 or 2 explanatory variables to determine which combination produced the best supporting model for each response variable. The full set of explanatory variables included: (a) number of fishing nets used during each fishing task, (b) tidal coefficient of the study area, (c) moon’s luminosity percentage, (d) environmental temperature (wet and dry) at the time of fishing, (e) fishing depth of the net, (f) CPUE, (g) total catch (kg) of the day (sum of all vessels’ fish catch), (h) number of boats fishing, and (i) boat’s catch (catch of the observed boat only).

The likelihood that a given model explained the response variable was assessed using second-order Akaike’s information criterion^35^ (AIC), where the model with the highest support has the lowest AIC value. All analyses were conducted in R^36^.

### Interview survey

We gathered the fishers’ perception about the relationship with SASLs and their language of valuation through interviews, participant observation, and a survey. We interviewed 23 fishers (50% of total fishers living in the area), all belonging to different vessels. The interview had both 35 open and 16 closed questions (Table S1). It gathered general socio-demographic information as well as sought to understand and discuss the conflict occurring between the SASL and the artisanal fishing industry from the fishers’s point of view. It is important to highlight that this particular fieldwork was done within a broader collaboration between INVASAL and La Barra fishing village^7,8^. INVASAL researchers have collected social and biophysical data during the last six annual fishing seasons and maintained collaborative relationships with the community. This study has to be seen not as the direct result of this particular field trip but as part of a longer knowledge and mutual collaboration.

Interviews covered the topics of: (1) interaction between SASLs and artisanal fishers (number of SASLs sightings per trip, methods used to chase away the SASLs, SASL mortality during fishing operations, changes in interactions over time), and (2) knowledge of the biology of SASLs (especially focusing on the ecological role of the SASLs in the ecosystem); understanding that knowledge is not formal information (i.e. not necessarily scientific knowledge) but rather practical rationalities (understood as the ability to evaluate the context and act properly to obtain results regardless theoretical knowledge^37^) held and circulated within the community. We did not test the actual knowledge, but rather asked for their self-evaluation of the level of knowledge about some issues. All the experimental protocols were approved by the Ethics Committee of the Universidad de Valparaíso. Also, all the interviewed fishers were informed and consented to participate in this survey.

To analyze the relationship between fishers and SASLs within a broader relationship with nature, the survey included a pilot set of Likert scales about the perceptions and views about nature. Likert scales are general surveys that analyze people’s feelings or attitudes with the objective of solving a technical problem^38^. This scale includes a series of statements about people’s degree of agreement (Table 1). The underlying idea in this scale is that the attitude of a subject or group is expressed through the evaluations of the presented items^39^, with five degrees of agreement; from strongly disagree (1) to strongly agree (5). In particular, we proposed a set of three Likert scales, designed to measure the fishers’ valuation language of nature. Scale 1, an instrumental view of nature, understanding the value of fish and the river itself as economic resources; Scale 2, a conservationist view of nature, which focused on environmental and ecological views whilst not accounting for economic benefits for the community; Scale 3, reflecting an “environmentalism of the poor” view, including sentences that acknowledge rights to utilize nature and the river, and sentences that link social wellbeing with a respect for nature. A deeper statistical analysis is required in order to validate the Likert scales. Our first step was to validate the scales, to see if these groups of sentences accurately measure these distinctive dimensions in a consistent way. It is expected that all sentences that measure the same dimension will form a cluster in a hierarchical conglomerate analysis using a Ward method dendrogram. However, the dendrogram did not form the expected clusters, and some adjustment was needed to obtain consistent scales. We will discuss an important modification of the environmentalism of the poor scales, affecting our original assumptions. We used Spearman correlation to relate the Likert Scales with the socio-demographic variables of (1) age, (2) level of education, (3) monthly income from fishery activities, (4) family monthly income, and (5) years dedicated to fishing. All statistical analyses of interviews and surveys were performed in SPSS.

**Table 1:** Original and modified Likert scales.

Originally proposed Likert scales	Modified Likert scales
**Scale 1. Instrumental view of nature**	
S02: I consider salmon only as an economic resource	S01: I think that all living beings in the river have the right to exist (inverted)
S07: I consider all fishes only as an economic resource	S02: I consider salmon only as an economic resource
S08: There are too many SASLs, you have to get them out of the river anyway	S07: I consider all fish only as an economic resource
S10: I think salmon have benefited the river and thevillage	S08: There are too many SASLs, you have to get them out of the river anyway
	S10: I think salmon have benefited the river and the village
**Scale 2. Conservationist view of nature**	
S01: I think that all living beings in the river have the right to exist	S03: I think only the common native species of the river should be protected
S03: I think only the common native species of the river should be protected	S04: I worry more about taking care of the river than the economy of the village
S04: I worry more about taking care of the river than the economy of the village	S09: I am not worried about the negative effects of the arrival of salmon (inverted)
S06: There should be more concern about the negative impact of salmon arrival	S11: I think that the livelihood of the village is more important than caring for the river (inverted)
S09: I am not worried about the negative effects of the arrival of salmon (inverted)	
S11: I think that the livelihood of the village is more important than caring for the river (inverted)	
S13: I think the SASLs should be protected	
**Scale 3. Environmentalism of the poor**	
S05: I am willing to fish only what is necessary so that the fish can reproduce	S05: I am willing to fish only what is necessary so that the fish can reproduce
S14: The economy of the village benefits when we take care of the river	S14: The economy of the village benefits when we take care of the river
S15: It is important to take care of the river because that way we all earn more	S15: It is important to take care of the river because that way we all earn more
S16: We have to look for the way we can live with the SASLs	

## Results

### Attack behavior of SASL to gillnet-captured Chinook salmon

The presence of SASLs was recorded in 20 out of 22 (90.9%) fishing trips (Table 2), with an average of 3.6 ± 1.9 individuals per trip. Interactions between SASLs and the fishing operation were observed in 7 of the 20 trips with SASLs present (35%) (Table 2). The types of interactions recorded included damage to fishing gear (one event), damage to the catch (four events), and perturbation of the fishing operation due to the presence of SASLs (two events). SASL interactions resulted in seven fish (~ 70 kg) damaged of a total catch of 2815 kg (2.5%) during the study.

**Table 2:** Dataset of variables registered during fishing activities.

Presence (P)/absence (A) of sea lions	Number of sea lions present	Presence (P)/absence (A) of interaction	Number of sea lions interacting	Moon's luminosity	Tidal coefficient	Environmental temperature (°C)	Number of boats	Number of fishing nets	Depth of fishing nets	Fleet total catch (kg)	Boat’s catch (kg)	CPUE
P	5	P	2	0.8	82	11.4	15	2	7	528.5	28.5	0.02
A	–	–	–	0.7	69	4.6	20	1	10	274.8	20.3	0.02
P	6	A	–	0.6	58	11.6	4	1	3	28	0	0.00
P	6	A	–	0.5	49	13.0	18	1	4	298.8	0	0.00
P	5	A	–	0.4	46	9.3	28	1	7	3495	218	0.34
P	2	A	–	0.3	49	10.0	25	2	7	8038	452	0.21
P	6	P	1	0.2	55	12.1	29	1	7	4035	20	0.08
P	4	P	1	0.1	62	11.9	28	1	2	13,814	176	0.28
P	3	A	–	0.1	68	9.2	14	2	5	1594	10	0.01
P	1	A	–	0.0	74	13.6	19	3	7	2712	20	0.01
P	1	P	1	0.0	74	13.6	19	1	7	2712	54	0.30
A	–	–	–	0.0	78	13.0	24	3	7	16,295	900	0.40
P	4	A	–	0.0	80	14.8	25	1	5	2305	43	0.04
P	3	A	–	0.0	79	10.6	22	2	7	1929	117	0.06
P	1	A	–	0.1	75	10.2	15	1	5	1644	3	0.02
P	4	A	–	0.1	69	12.1	26	1	7	625	29	0.02
P	4	A	–	0.1	69	12.1	26	1	5	625	0	0.00
P	1	A	–	0.3	55	6.8	3	1	7	59	41	0.06
P	1	P	1	0.3	55	6.8	3	1	7	59	13	0.02
P	3	P	1	0.4	48	13.8	10	1	10	1006	152	0.07
P	6	A	–	0.5	45	9.5	26	3	4	3240.3	248	0.03
P	6	P	2	0.6	48	13.0	28	2	9	7108	270	0.18

### Factors explaining the presence of SASL and interactions with fishing tasks

**Figure 2:**
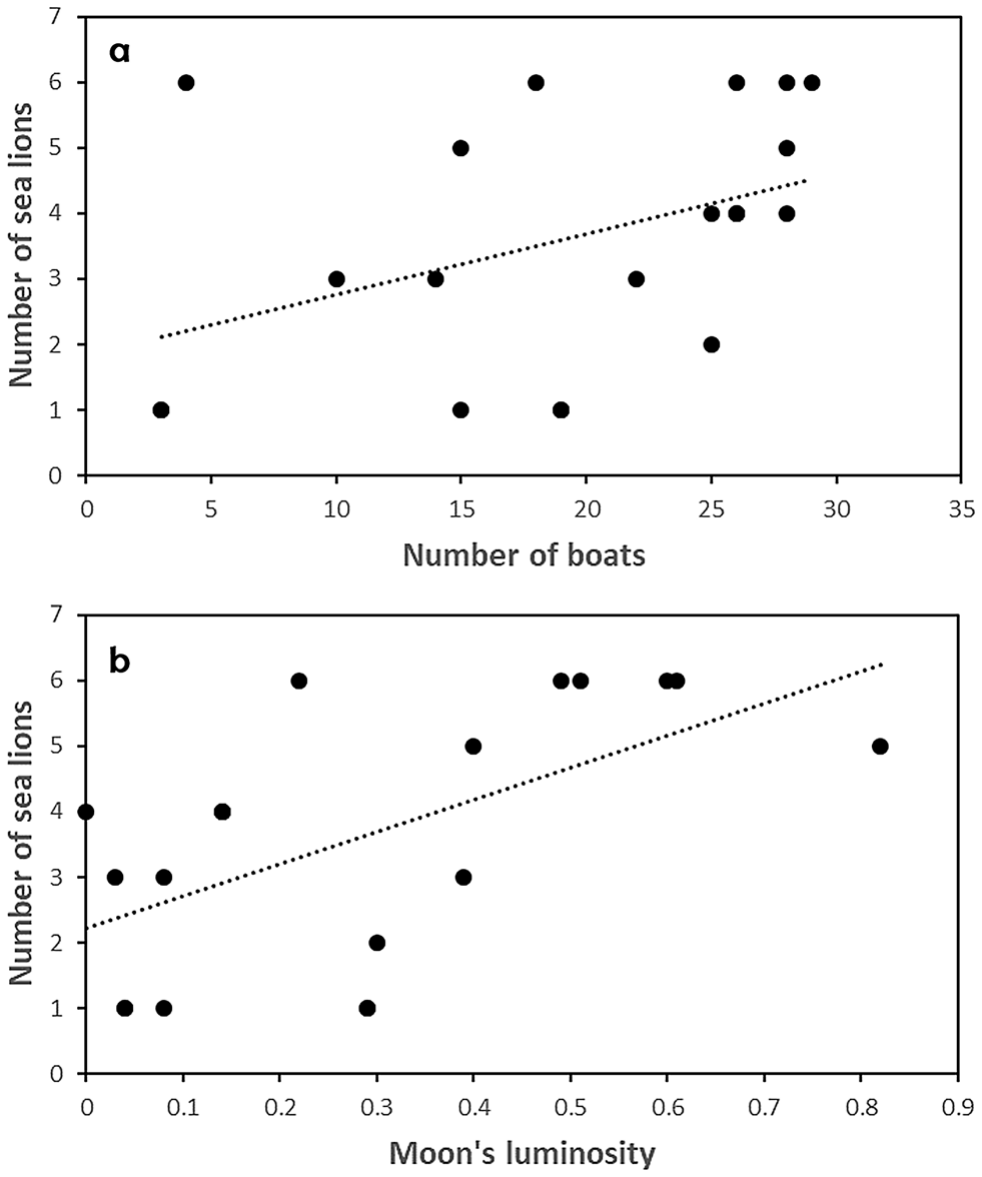
Relationship between (**a**) number of boats operating and number of SASLs, and (**b**) moon’s luminosity and number of SASLs.

**Figure 3:**
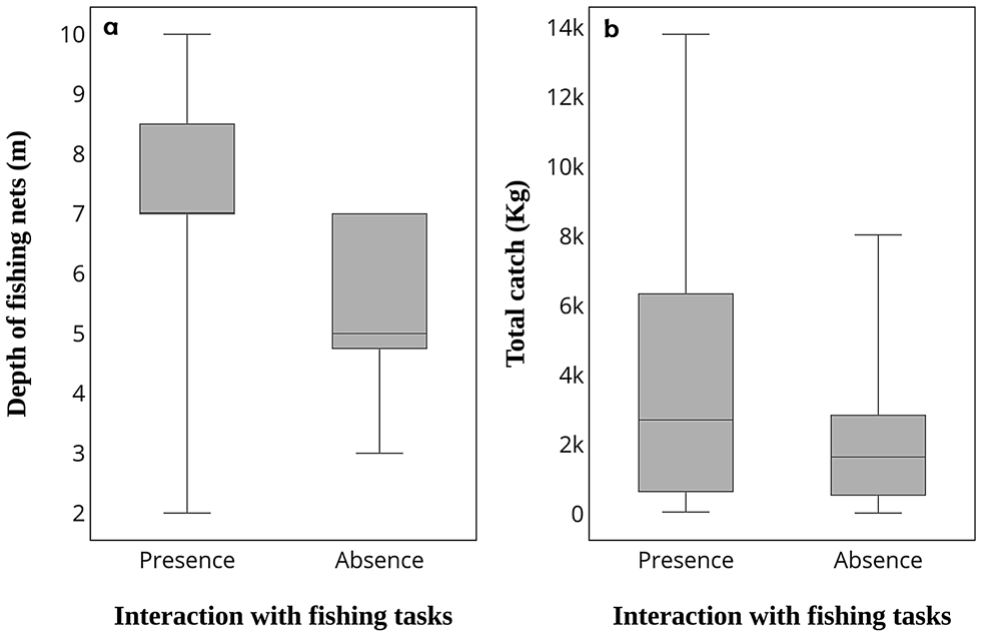
Relationship between the occurrence of interactions with the fishing tasks and (**a**) the depth of the fishing nets, and (**b**) the total salmon catch.

The best supported model to explain the number of SASLs observed in the fishing area was the one including number of boats and moon’s luminosity (Table 3). Both number of boats (GLM, *Z* = 2.305, *P* = 0.021) and moon’s luminosity (GLM, *Z* = 2.979, *P* = 0.003) correlated significantly with the number of SASLs present (Fig. 2). The model with the highest support explaining the occurrence of an interaction between SASLs and the fishing operation included depth of the fishing net and total catch of the day (Table 3). Interactions occurred more often when fishing nets were deeper and the total catch was higher (Fig. 3). However, none of these variables alone were statistically related to the occurrence of an interaction (GLM, Depth: *Z* = 1.742, *P* = 0.081; Catch: *Z* = 1.726, *P* = 0.084).

**Table 3:** Predictive models tested to explain the variation in the number of SASLs observed during the fishing activities and the occurrence of interaction with the fishing tasks.

Response variable	Tested model	AIC	ΔAIC
Number of SASL observed	Luminosity + boats	75.91	0
	Luminosity	79.66	3.75
	Luminosity + tide	81.24	5.33
	Boats	82.44	6.53
	Tide + boats	82.51	6.60
	Tide	83.74	7.83
	Null model	84.05	8.14
Occurrence of interaction	Depth + catch	25.81	0
	Depth	27.25	1.44
	Depth + CPUE	27.53	1.72
	CPUE	27.71	1.90
	Null model	27.90	2.09
	Catch	28.05	2.24

### Fishers’ perception of the conflict

The survey included responses from 22 male fishers and 1 female fisher reflecting the gender bias of the activity. The sample contained a balance of young, less experienced fishers, and older, more experienced fishers: 56% were under 45 years old, and 39% were over 60 years old. Sixty five percent of those over 60 years old had worked on fishing boats for more than 25 years. The level of formal education was generally low: 56% of interviewees only completed primary school. Most interviewees (61%) were full-time fishers; the others had additional jobs. Most interviewees (74%) had an income below the national minimum wage, which is about US$460 in 2021.

A total of 87% of the interviewed fishers considered SASL interactions very important to their activity (in a simple scale of 5 degrees, from not important to very important). In order to clarify what “very important” means, this was followed by an open question, in which their responses were very consistent. Seventeen respondents (77% of the sample) described the interaction as “*bad*”. Also, other six respondents used negative attributes such as *“problematic”, “being enemies”, “conflictive”, “difficult”,* and even *“hate”*. No positive or neutral attributes were used to describe the relationship. Some of the interviewees provided a more detailed view of this perception:

“*the beast is intelligent, it knows were to be and when to attack… it is very intelligent*”* [“si si el bicho es inteligente, sabe dónde ´ponerse y cuándo atacar, es muy inteligente”]* (Pedro, fisher)

“*here we are, repairing the sea lion mess… what else can we do?… , it is what it is, and we are used to do that*” [“*aquí estamos, usted nos ve, arreglando la cagaita del lobo… pero ya que le vamo’ a hacer, es así la cosa, ya estamos acostumbrados*”] (Juan, fisher)

*“the problem is when the sea lion catches one salmon, and how much does a salmon cost?… they are like 15 kilograms… and you don’t find buyers for a bitten salmon* [*“el problema es el lobo que te pesca uno y ¿cuánto vale uno si son como 15 kilos?, y después está el problema de que a veces no hay compradores, pero uno ya sabe ya”*] (Juan, fisher)

Most of them (96%) consider that the number of interactions has increased overtime, and 39% acknowledge that these interactions would end if a SASL culling quota is assigned to fishers.

**Figure 4:**
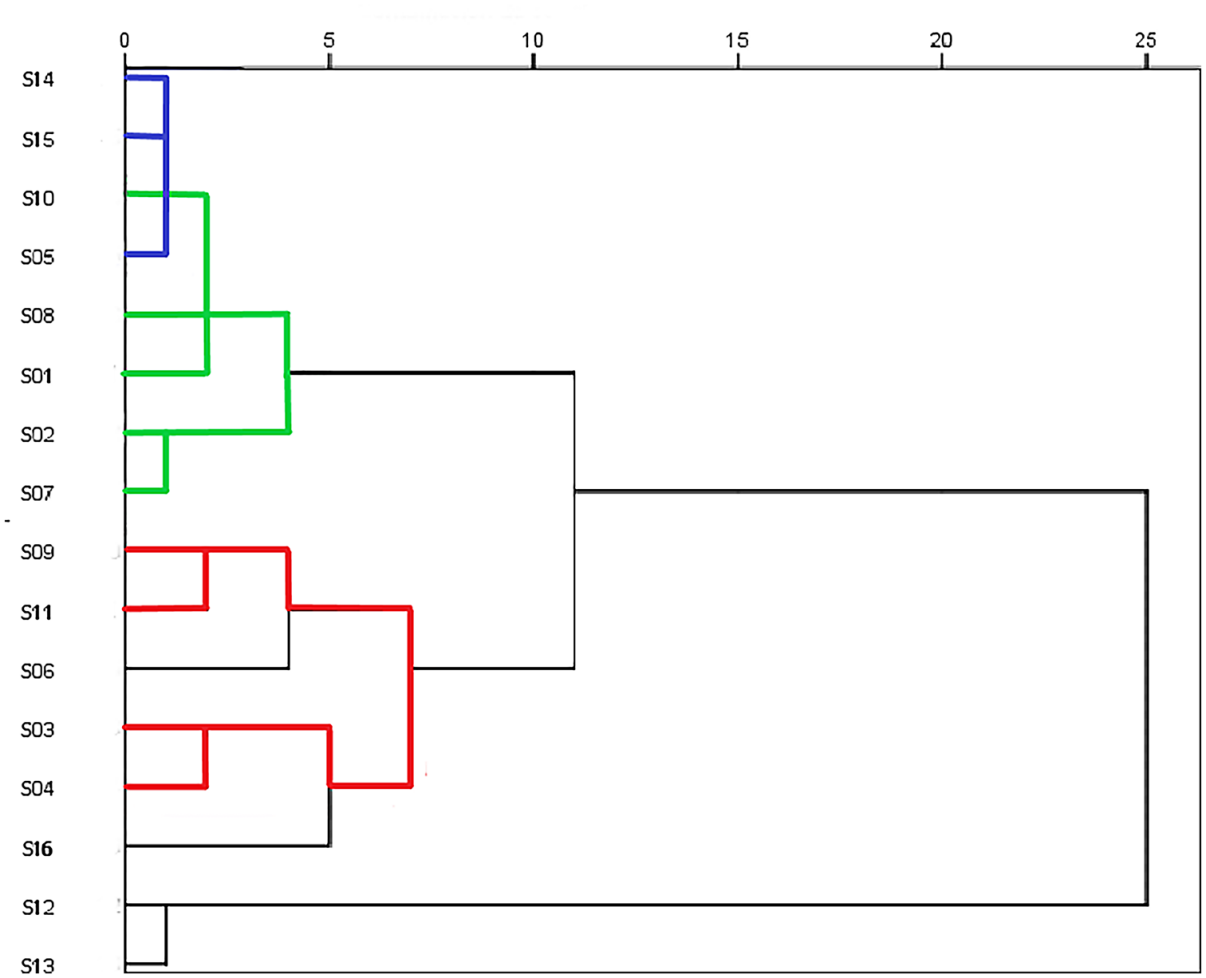
Ward method dendrogram. Combination of re-scaled distance clusters. Green lines indicate an instrumental view of nature, red lines indicate a conservationist view of nature, and blue lines indicate the environmentalist of the poor.

Analyzing the Likert scales about “valuation language”, two clusters grouped most of the sentences (Fig. 4), prompting a reevaluation of the original scales, according to what was empirically found in the field (Table 1). The first cluster groups the sentences that express conservationist rationality (S03, S04, S16) and two sentences that express an instrumental one, but inverted (S09 inv. and S11 inv.). The second cluster shows an interesting mix between two rationalities: an instrumental one viewing fishes as resources (S02, S07, S08, S10), and some variables of “environmentalism of the poor” (S05, S14, S15) such as “I think the economy of the village benefits when we care for the river” as well as one inverted “environmentalism of the poor” sentence (S01 inv.). Therefore, instrumental views appear to coexist with some ecological views about the whole river, particularly views that link local economic and environmental wellbeing. However, they do not appear to coexist with more substantive aspects of the “environmentalism of the poor”, in particular, statements that acknowledge a right for natural beings and SASL to exist (S01 and S16). This instrumental view also does not relate with a conservationist one.

Based on this analysis we modified the original scales (Table 1). We removed some items that did not correlate with the other sentences of the same scale. We also moved some items between the conservationist and instrumental scales, because they worked better statistically as an inverted statement. And finally, although the instrumental and “environmentalism of the poor” sentences seemed to correlate, we decided to keep them as distinct indices, although slightly modified, removing all the sentences that did not correlate from Scale 3. It is important to note that these changes transformed the meaning we gave to Scale 3. Therefore, it can no longer be named “environmentalism of the poor” as defined by Guha and Martinez Alier^27^, but rather becomes more similar to the popular view of “sustainable development”, that is a compromise between environmental conservation and the popular economic wellbeing.

The analysis of the Likert scales about environmental rationalities shows that a “popular sustainable development” view is commonly held in the sample (4.8 out of 5). We found that the instrumental rationality (4.0) view followed in frequency, whereas conservationist rationality (3.2) had the lowest value, and with a higher dispersion in the sample.

The correlation between the three Likert scales and the socio-demographic variables is shown in Table 4. We did not find a statistically significant correlation for any variables in relation to the popular sustainable development. The productivist-instrumental rationality (i.e. nature is seen as a resource to be managed in order to obtain some benefit sustained over time) showed correlations with age (the older, the most instrumental), years in fishing (the more years, the most instrumental), and a trend with educational level (the more years of formal education, the less instrumental). The conservationist rationality did not show any significant correlation, as an index or as individual sentences. In spite of this lack of significance (probably due to the small sample size), in the qualitative interviews all the younger fishers with more years of formal education (n = 8) expressed more conservationist views.

**Table 4:** Correlation between scale and sociodemographic variables.

Scale/variable	Age	Study level	Monthly income from fishing	Family monthly income	Years dedicated to fishing
Conservationist	− 0.283	0.149	0.091	0.264	− 0.271
Instrumental	0.551**	− 0.350	0.017	− 0.114	0.482*
Popular sustainable development	0.077	− 0.260	− 0.167	0.202	0.141

*p < 0.05, **p < 0.01, ***p < 0.001.

## Discussion

Our study addressed the interaction between the SASL and the operation of the Chinook salmon fishery in the Toltén River, supported by a non-native and invasive species found populating many rivers and adjacent sea of Chile^6^. Our results demonstrate that the interaction between SASLs and the small-scale fishing communities of Caleta La Barra vary depending on different factors, both operational, such as the number of boats, and environmental, such as moon luminosity during the fishing operations. We were able to determine common patterns among fishers that allow us to establish different social profiles that shape their relationship with the SASL and also their relationship with the natural environment in which they live.

Despite the frequent presence of SASL during fishing operations (> 90%), we assessed that only 35% of them constituted interactions. This frequency of interaction is lower than recorded by other studies in this species^18,20,21^, but higher than in others^17^. These differences indicate that there is a high level of variability in the frequency of interactions between the SASL and the artisanal fisheries, both at spatial^20,21^ and temporal^17,18^ scales. According to de María et al.^40^, these differences can be explained by different factors such as the season in which the study was carried out, the productivity of each area, the fishing gear used and the captured species, among others. As the Chinook salmon is only fished during the austral summer months (January and February), it was not possible to analyze potential temporal variations in the frequency of interactions in this study.

A positive relationship was observed between the number of SASL and the number of boats operating. A similar relationship was reported by Goetz et al.^19^, who observed groups of SASLs following boats during fishing operations. This can be explained by a number of factors. First, Szteren and Paéz^15^ reported that SASL in Uruguay are able to recognize the sound emitted by the boats during a fishing operation, and that is an indicator of prey availability (“dinner bell” effect). Second, it is possible that some individuals have learned to feed during fishing operations, specializing in a type of prey or in a particular feeding strategy associated with fishing operations^41^. Likewise, due to the general decrease in resource availability due to overexploitation in Chile^42^, SASLs could be attracted to fishing activities, associating a higher number of boats with greater availability and easy-to-capture prey^19,43^.

We also found a positive relationship between the number of SASLs and the moon’s luminosity. Nights with high lunar luminosity (full moon) are associated with the largest tidal ranges of the month. These tides are related to a high productivity due to the formation of tidal fronts characterized by the abrupt difference in temperature, oxygen and fluorescence, commonly observed during spring tides, increasing the concentration of zooplankton and therefore attracting more predators^44^. This could result in greater resource availability. Largest tidal ranges may be also linked to influx of returning Chinook salmon in higher numbers than the rest of the month (authors’ unpublished results). However, it cannot be ruled out that the better visibility provided by the increased luminosity would have allowed the observer to count a greater number of SASLs around the boat^16,19^.

Although non-significant, we found that the interactions between SASLs and the fishing operation occurred more often when fishing nets were deeper and the total catch was higher. A relationship between the occurrence of interactions and depth could be related to the time the net is underwater. At greater depths the time of hauling increases, which in turn increase the opportunity for the SASL to interact with the boat. This trend is opposite to what was found by other authors in other species^45^ who observed more interaction close to the surface as a learned strategy to reduce energetic demands. However, in our study the maximum depth of a fishing net was 10 m which is much lower than the depth SASLs usually dive during their foraging trips^46^. With respect to the relationship between the occurrence of interactions and total catch, different studies have demonstrated that fishing operations and seals and sea lions coincide in areas where the resource is more abundant^15,47^, and thus the number of SASL raiding the nets increases when there are more fish caught in the net and easily accessible.

We found no relationship between CPUE and the interaction of SASL during fishing operations, i.e. no variations in the standardized catch of Chinook salmon per haul were recorded regardless of interactions with SASL. Similar results have been identified by other authors in studies of gillnet and purse-seine fisheries in other areas, both in Chile and elsewhere for this same species^18,19,26,40^. This lack of relationship could be explained by a number of factors. Firstly, the number of fish consumed by SASLs in the fishing gear is not high enough to generate differences in the CPUE at the fishery scale^15,18,20^. Secondly, and as mentioned before, SASLs and fishing operations coincide in areas where the resource is more abundant. Therefore, and even if an interaction with the SASL was reported, the CPUE was maintained or was higher in areas where SASLs are present, in comparison to areas where this predator is absent^15^. Finally, the volume of fish catches is affected by additional factors besides the presence of SASLs, such as environmental conditions, the presence of other predators, and the abundance of resources in each area, and therefore do not exclusively depend on interactions with the SASL^40^.

South American sea lions are frequently blamed for causing significant impacts to the economy of local fishers^26^. However, at the scale of the whole fishery, damage to catch from SASLs interactions was recorded in only four of 22 fishing events and in 2,5% of the total catch, suggesting that damage is smaller than perceived by fishers. It is important to note, however, that the assessment of damage by SASLs in this study is conservative, since we do not consider fish that could be wholly removed from the net, which can increase the total biomass lost by SASLs. Competition for fishing resources between SASLs and artisanal fishers has been largely documented in different areas and situations and will likely exacerbate negative perception about this mammal and an obstacle to fishing operations^17,24^. In a recent study, Oliveira et al.^26^ demonstrated that the actual economic losses caused by sea lions to the local fishery in Brazil are much smaller (0.8–3% of the productivity of the monitored boats for the analyzed year) compared to the large damage perceived by the fishers. This perception extends to other cases of human-big marine carnivore interactions and competition during fishing activities^48,49^. Therefore, it is possible that the observed negative perception about SASLs is independent of a particular event, but it rather relates to the continuous presence of SASLs in the area, and also to the general perception that the SASLs can eat hundreds of fish over the course of one interaction^26^.

However, it is important to note that economic losses could be relevant on a boat-to-boat basis, and this clearly contributes to exacerbate a negative perception towards SASLs. In the case of the Chinook salmon fishery from Toltén River, we quickly appraised that SASL interactions may result in significant losses on a boat-to-boat basis. Ex-vessel price of Chinook salmon has been on average CLP$3500 per kg (USD$5; currency rate at 27 April 2021), suggesting that a 10–15 kg salmon may be worth CLP$35,000–53,000 (USD$50–76). Boats with a damaged catch by SASL lost up to 11% of their revenue during the duration of the survey. Considering fishers’ sole reliance on a short fishing season^8^ and the overexploitation of alternative, native fishery resources^42^, fishers’ perceptions are likely to be negative towards SASL given these economic losses.

The negative perception of SASLs by fishers needs to be understood within the context in which nature has been historically and socially altered. Since Chinook salmon was successfully introduced around 25 years ago^50^, both human fishing practices and the behavior of SASLs have changed in important ways. Fishing has moved from a year-round communitarian activity performed in the open sea, focused on abundant small native marine species, to a mostly familiar activity, performed in the estuary during a certain period of the year and focused on a big and profitable catch. The SASLs have also learned to feed on this new species and changed their predatory practices, moving from the sea to the river, and predominantly predating on salmon in the short period in which they arrive in the estuary. There is a mutual coproduction^31–33^ process in which these three parties—salmon, fisher, and SASLs—have each modified their behaviors and condition of reproduction and existence. The salmon colonized a new habitat, fishers learned to fish this new attractive species, and the SASL also learned to prey on this new species, colonizing a new space in the estuary, following fishing boats. This coproduction has presented new opportunities for the human population, as they have been able to exploit a new economic resource that has revitalized the economy of the town, but it has also refueled a long-standing conflict between humans and SASLs. Furthermore, these changes in fishing activity may have increased the perceived interactions with SASLs and the damage they cause. Moving fishing operations from the sea to the smaller and calmer estuarine waters makes the sea lion more visible. Also, a fishing based on a big (~ 15–20 kg each) and profitable Chinook salmon species, makes any sea lion attack more damaging than former fishing practices in which SASLs took one or two small fish from big net hauls.

In order to address the negative perception of SASLs amongst artisanal fishers, we framed the discourse about SASLs within a broader discourse with nature using the concept of valuation language from Martinez-Alier^51^. Using Likert scales we observed that, contrary to what was expected and seen in other contexts, fishers do not demonstrate a view in line with the “environmentalism of the poor”. However, they did possess a view that values sustainability, though not in a conservationist context that would be expected from a community that rely on natural resources for their livelihood, but it is not purely instrumental either. This leads us to rethink the Martinez-Alier^51^ categories in order to describe the combination that was found among fishers in this study: a mix between elements of utilitarian rationality, viewing fish solely as a resource, and elements of a deep caring for the river and sea life, linking local economics with environmental wellbeing. However, this view did not include some of the more substantive and political aspects of an “environmentalism of the poor” perception, such as the right for all natural beings to exist. This discourse was better described as part of the “popular sustainable development” view, as the sustainable development paradigm is based more on an economic, rather than ecological, rationality that transforms ‘nature’ into ‘environment’ and resources^52^. However, in this case, caring for the river and sea, and economic wellbeing were seen as mutually necessary in a discourse that is accompanied by the respect of natural forces as being important to the life and wellbeing of the community.

We also found that most fishers, from all socio-demographic characteristics, hold this “popular sustainable development” view. This was followed by instrumental rationality (4.0), held mostly by older fishers with lower levels of formal education. These results are consistent with those found in other studies^24,53,54^, in which older fishers have the most negative attitude and perception of SASLs because of the several negative encounters with these species^24^. This commonality of the popular sustainable development view, accompanied by a more conservationist view held by younger fishers with more years of formal education, opens up the possibility for a better relationship among Chinook salmon, fishers, and SASLs, and a better coexistence. We hypothesize that it is possible to make local effective governance changes to improve this coexistence^55^, for example, by changing the fishing practices to modify the SASL behavior. Currently, fishers process the fish and dispose of the waste in the river where they fish. In their own words this practice “domesticate” SASLs, and the community is making agreements to move this practice away from the shore. It also seems that SASLs follow the boats as they have learned that boats mean availability of prey. This requires improved governance systems to redistribute fishing places within the river, as fishers already partially and informally do it. Also, it is recommendable the implementation of a long-term education program to the fishing community that include, among other issues, the critical role of this marine top predator on the trophic webs and consequently the negative impacts of their removal^18,24^. Actions like these could help to move from a paradigm of “defending the fishing from the SASLs” to a better local understanding of the relationship between Chinook salmon, SASLs, and human behavior. In recent years, due to the important economic opportunity that Chinook salmon has meant for the community, young fishers with more years of formal education, and a more sustainable and conservationist view of fishing, have returned to La Barra to occupy leadership roles in the community. We cannot be sure how this leadership will evolve as they grow older and more experienced, but the formal education and experience backgrounds they hold separate them from their parents and may anticipate a different trajectory. We speculate that this is a step in the right direction at resolving SASL-fisheries conflicts.

## Supplementary Information

Supplementary Table S1.
