# Supplementary material for: Fishers' perception of the interaction between the South American sea lions and the Chinook salmon fishery in southern Chile
Source: Sci Rep. 2021 Jul 14;11:14463. doi: 10.1038/s41598-021-93675-x (PMC8280164; doi:10.1038/s41598-021-93675-x)
Supplement: Supplementary file 1 — Supplementary Table S1. [file 41598_2021_93675_MOESM1_ESM.docx]

**Fisher’s perception of the interaction between the South American sea lions and the Chinook salmon fishery in southern Chile**

**Running title: Interactions between the South American sea lion and the salmon fishery**

Sanguinetti, M^1,2^., B. Cid^3,4^, A. Guerrero^1,4^, M. Durán^3,4,5^, D. Gomez-Uchida^4,6^ & M. Sepúlveda^1,4,*^

Table S1. Open and closed questions applied during the interviews to fishers from La Barra village, to gather the fishers’ perception about the relationship with the South American sea lion and their valuation language

| **Closed questions** | **Open questions** |
| --- | --- |
| - Occupation on the boat - Number of crew on the boat - Type of boat - Fishing target species - Days of the week when you do fishing activities - Sociodemographic questions (age, civil status, family group members, educational level, occupation) - Name of the boat - Fishing gear characteristics - Indicate the level of importance that you give to the interaction with sea lions (scale) - Name 3 impacts that the arrival of the salmon has had on the river and the village - Names 2 positive and 2 negative things about the arrival of salmon to the sector - Name the 3 main points of conflict since the arrival of the salmon - How many kg. per species are lost due to the interaction with SASLs - How much is the estimated cost to repair damaged equipment? - How much extra time do you have to dedicate when sea lions damage the gear? - What is the diet composition of the sea lions? mention 3 options in order of priority. | - What kind of fishing gear do you use? - Product marketing method - Fishing schedules - How do you get the product for sale? - Number of people associated with the boat that delivers the product - Average monthly income considering only fishing - Do you carry out other activities besides fishing that generate income? - Monthly income considering fishery and other activities - Is there another kind of income in your family group? - How much is the income? - How many hours a day do you dedicate to fishing? - Indicate the local actor who should manage the river - Do you extract another product besides salmon? - How many years have you dedicated to fishing? - How often do you interact with sea lions in your fishing trips? - What types of interaction do you observe that sea lions have when fishing? - At what haul phase does the interaction with the sea lions occur? - How do you see the relationship with sea lions? - Has the interaction of SASLs with artisanal fishermen increased over time? - What do you think could be the causes of the above? - What do you consider are the main factors that attract sea lions? - In your opinion, is the community informed of the problems that exist between fishers and sea lions? - In your opinion, how do you deal with sea lions interactions while carrying out artisanal fishing activities? - Would you be interested in learning a method to drive away them? - Is the fishing gear owned or rented? - Can you identify the sex and age of a sea lion? - Do you know how many years a sea lion lives? - Do you know the amount of time they spend with their young? - Without considering the interaction with fishing, can you indicate some appreciation of the SASLs - What kind of damage does the interaction between SASLs and artisanal fisher? - Is there mortality of sea lions during fishing? - Do you have any training or knowledge in how to deal with the SASLs in the fishing area? - What do you understand by the term ecosystem? - What do you think is the relevance of the sea lion in the ecosystem? - Mention any predator of the sea lion |
